# Periventricular heterotopia and white matter abnormalities in a girl with mosaic ring chromosome 6

**DOI:** 10.1186/s13039-015-0162-3

**Published:** 2015-07-26

**Authors:** Satsuki Nishigaki, Takashi Hamazaki, Mika Saito, Toshiyuki Yamamoto, Toshiyuki Seto, Haruo Shintaku

**Affiliations:** Department of Pediatrics, Osaka City University Graduate School of Medicine, 1-4-3 Asahi-machi, Abeno-ku, Osaka, 545-8585 Japan; Institute for Integrated Medical Sciences, Tokyo Women’s Medical University, 8-1 Kawada-cho, Shinjuku-ward, Tokyo, 162-8666 Japan

**Keywords:** Ring chromosome 6, Brain MRI, Periventricular heterotopia, White matter abnormality, Array-comparative genomic hybridization, Small for gestational age

## Abstract

Ring chromosome 6 is a rare chromosome abnormality that arises typically *de novo*. The phenotypes can be highly variable, ranging from almost normal to severe malformations and neurological defects. We report a case of a 3-year-old girl with mosaic ring chromosome 6 who presented with being small for gestational age and intellectual disability, and whose brain MRI later revealed periventricular heterotopia and white matter abnormalities. Mosaicism was identified in peripheral blood cells examined by standard G-bands, mos 46,XX,r(6)(p25q27)[67]/45,XX,-6[25]/46,XX,dic r(6:6)(p25q27:p25q27)[6]/47,XX,r(6)(p25q27) × 2[2]. Using array-comparative genomic hybridization, we identified terminal deletion of 6q27 (1.5 Mb) and no deletion on 6p. To our knowledge, this is the first report of periventricular heterotopia and white matter abnormalities manifested in a patient with ring chromosome 6. These central nervous system malformations are further discussed in relation to molecular genetics.

## Background

Ring chromosome 6 is a rare chromosome abnormality that arises typically *de novo* [1]. The phenotypes can vary from almost normal to severe malformations and mental defects [2]. The most common clinical features include intellectual disability, microcephaly, prenatal growth failure, retarded bone age, short neck, and typical facial anomalies [3]. As for brain abnormalities, hydrocephalus is the most important prenatal feature [4]. In addition to cortical atrophy and ventriculomegaly, absence of olfactory tract, agenesis or hypoplasia of the corpus callosum, aqueduct stenosis, and neuronal migration defects are also observed in previous reports [5].

Here, we report a case of mosaic ring chromosome 6 in a 3-year-old girl who presented with failure to thrive, microcephaly, and developmental delay and whose brain magnetic resonance imaging (MRI) later revealed periventricular heterotopia and white matter abnormalities. Neither of these central nervous system anomalies has been previously reported in association with ring chromosome 6.

## Case presentation

### Clinical description

The patient, a female, is the second child of healthy unrelated parents. Family history, including her 4-year-old sister, was unremarkable. This patient was born by means of caesarean delivery because of intrauterine growth retardation at the 39th week of gestation. Birth weight was 1,785 g (<−2SD), length was 43 cm (<−2SD) and head circumference was 29 cm (<−2SD). Apgar scores were 8 and 9 at 1 and 5 minutes, respectively. She was able to sit at 5 months, walk at 14 months. Although at age 3 years she couldn’t speak in two-word sentences, at age 3 years and 11 months she was able to speak three-word sentences with postpositions. She had a history of repeated febrile seizures.

**Fig. 1:**
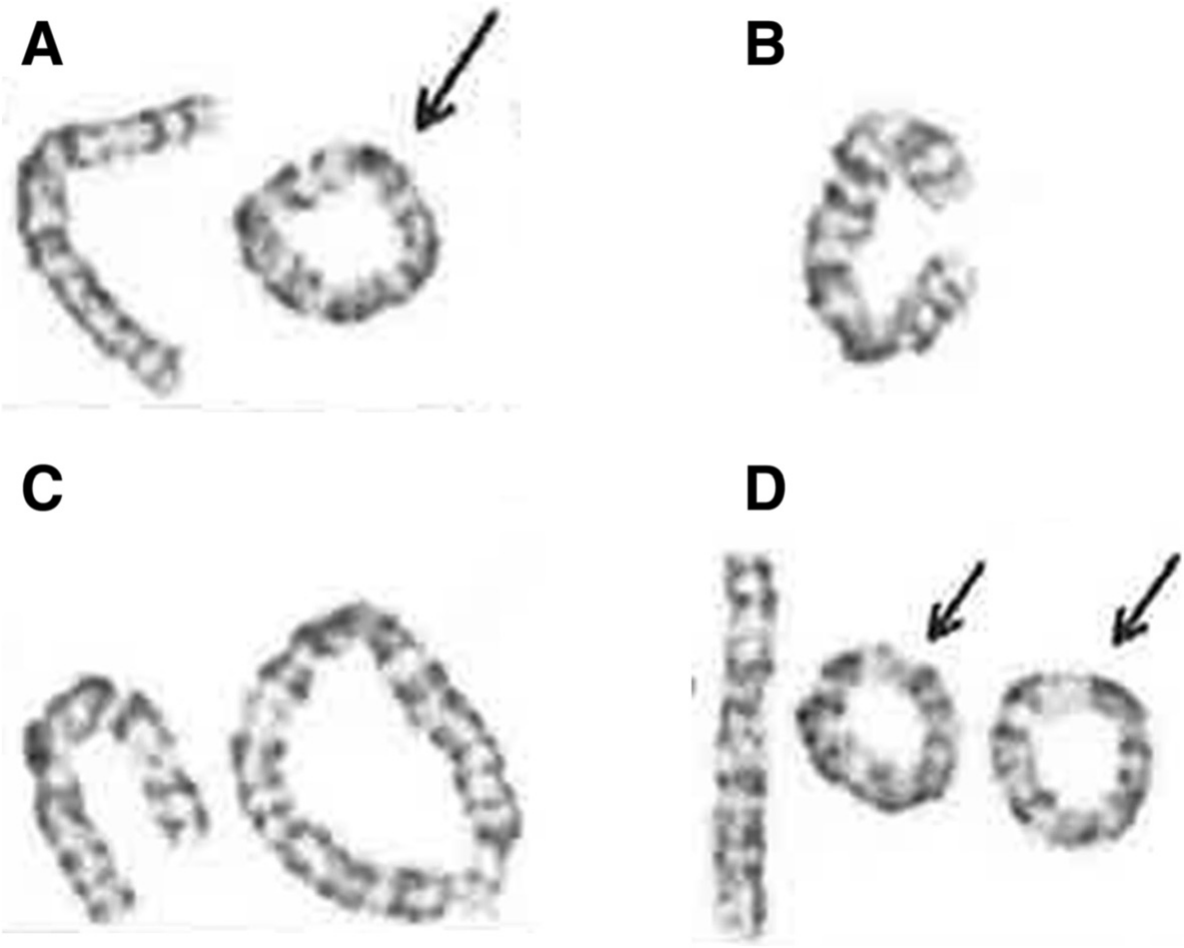
Chromosome G-banding result from the patient. The total number of cells counted was hundred. **a** Ring chromosome (67 %). **b** Monosomy 6 (25 %). **c** Dicentric ring chromosome (6 %). **d** Double ring chromosome (2 %)

At the age of 3 years and 11 months, her weight was 9,300 g (−3SD), length 89.7 cm (−2.4SD) and head circumference 41.3 cm (−4.4SD). Short stature prompted endocrinological investigations and karyotyping. Exaggerated growth hormone responses to arginine, levodopa and clonidine stimulation were within normal range, whereas karyotype analysis revealed mosaic ring chromosome 6 (Fig. 1). She presented minor dysmorphic features including full lips and epicanthal folds. Ophthalmological and otological including aural examination were normal, and ultrasound cardiac examination revealed no anomalies. Kyoto Scale of Psychological Development, which is a standardized developmental test for Japanese children, was performed at 3 years 10 months, revealing postural-motor skills of 3 years 1 month (subquotient 79), cognitive-adaptive skills of 2 years 6 months (subquotient 64), language-social skills of 2 years 9 months (subquotient 70), and overall developmental age of 2 years 8 months (developmental quotient 68).

**Fig. 2:**
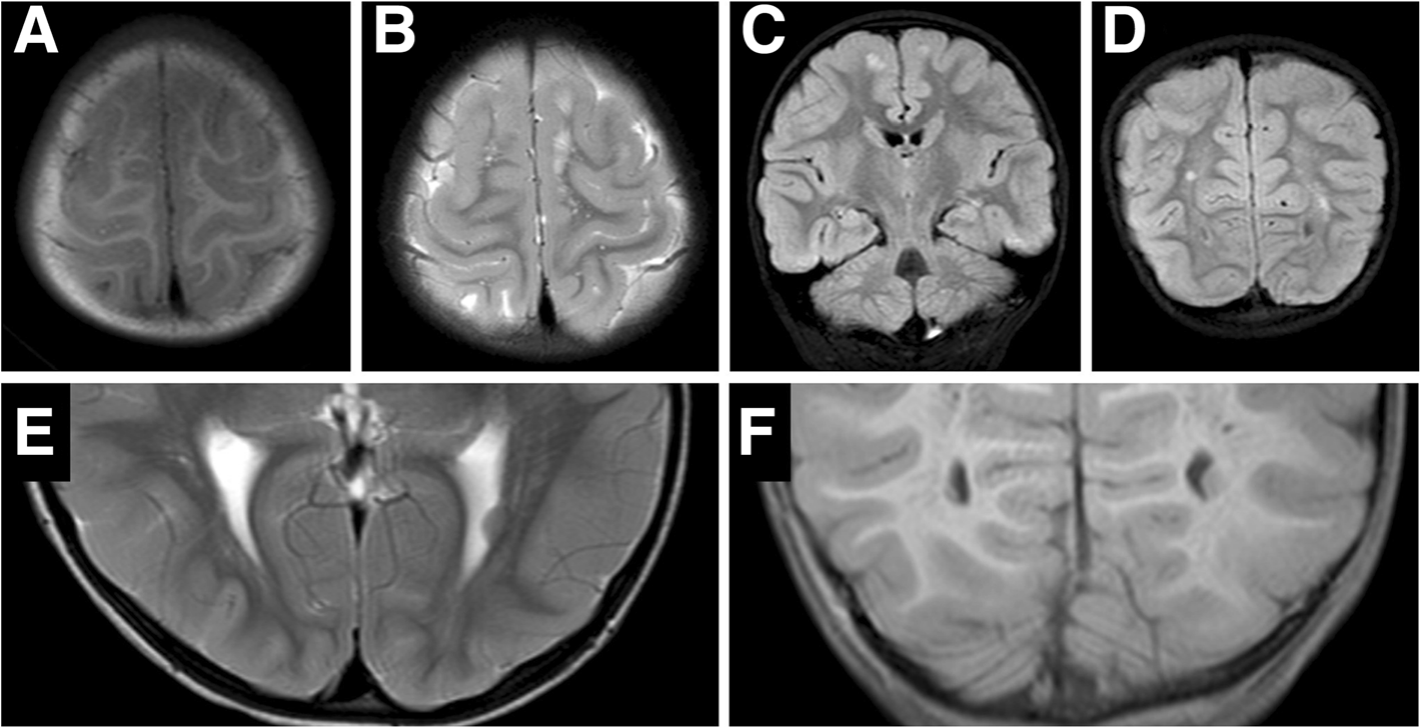
**a-f**: MRI imaging of the brain of this patient. **a** and **b** Axial T1W and T2W image, showing left parietal lobe. **c** Coronal T2W section showing white matter abnormalities in the bilateral parietal lobe. **d** Coronal T2W section showing white matter abnormalities in the bilateral occipital lobe. **e** and **f** Axial and Coronal FLAIR image, showing periventricular heterotopia on the bilateral posterior horn of the lateral ventricle

Cerebral MRI revealed periventricular heterotopia on the bilateral posterior horn of the lateral ventricle and white matter abnormalities in the bilateral parietal and occipital lobe. Neither hydrocephalus nor enlarged lateral ventricles were detected (Fig. 2). Electroencephalogram (EEG) during induced sleep showed poor normal sleep spindles and slightly irregular background activity in the bilateral occipital head regions. The possibility of tuberous sclerosis was also considered with respect to the observed periventricular nodules. Besides the past episodes of febrile seizure, she never had an epileptic attack, and anticonvulsant agent was never prescribed, ruling out the possibility of tuberous sclerosis. This study was approved by institutional review board (Osaka City University).

### Molecular and cytogenomic characterization

**Fig. 3:**
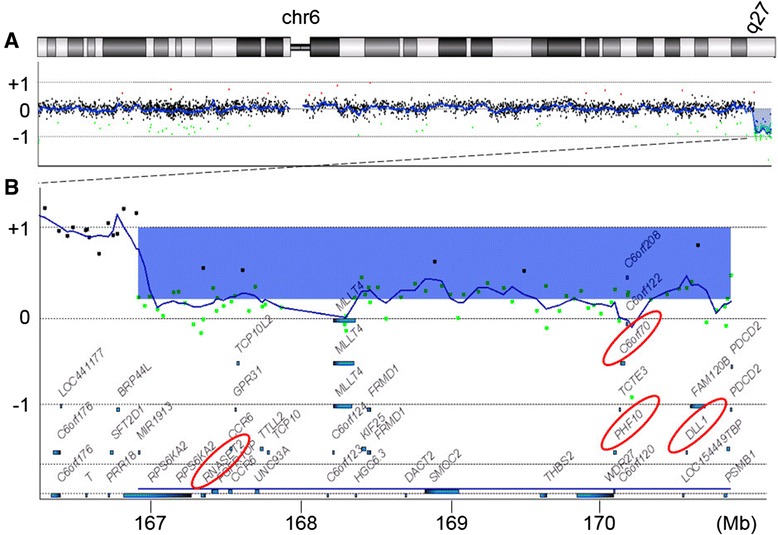
The result of array-CGH. Vertical and horizontal axes indicate the genomic location and signal log_2_ ratio, respectively. Dots indicate the location and the signal log_2_ ratio of the microarray probes. **a** Chromosomal view of the chromosome 6 shows a genomic copy number loss at q27. **b** Gene view expands the aberration region. The blue rectangle indicates and the blue bars indicate the aberration region and the locations of the genes. Two genes discussed in the text are marked by red oblongs. The mean log_2_ ratio of the aberration region is −0.780567, indicating mosaic deletion

This study was conducted following approval by the ethics committee at our institution. After obtaining the written informed consent from the patient’s family, blood sample was drawn from the patient. Chromosome analysis was performed on peripheral blood lymphocyte cultures. Standard G-bands analysis revealed mos 46,XX,r(6)(p25q27)[67]/45,XX,-6[25]/46,XX,dic r(6:6)(p25q27:p25q27)[6]/47,XX,r(6)(p25q27) × 2[2]. The microarray-based comparative genomic hybridization (array-CGH) analysis was performed as described previously [6] and revealed a genomic copy number loss at 6q27 (Fig. 3).

## Discussion

In general, ring chromosomes are formed by fusion of the distal ends in both arms and often lose genomic material of the terminal ends. The rings may result in clinical features mimicking terminal deletion syndromes. On the other hand, a patient carrying a ring chromosome 6 without terminal deletions has been reported with short stature and minor dysmorphisms but with normal psychomotor development [7]. This indicates that carrying a ring chromosome itself may lead to these clinical features. These prenatal and postnatal growth failures, also seen in the present case, are common features of any autosome ring chromosome. It has been suggested that this is due to the mitotic instability of rings, preventing normal cellular proliferations [8, 9].

Terminal deletion on 6q has been linked to certain brain abnormalities as well as neurological signs and symptoms. Based on a series of the array-CGH analysis, Eash et al. have suggested that the terminal region of chromosome 6q is a critical area for intellectual disability, hydrocephalus, abnormal corpus callosum, seizures, and hypotonia [10]. In the present case where the terminal of 6q27 was deleted, none of those clinical features was present except for intellectual disability.

A few studies have investigated the effects of terminal deletion on 6p. In an adult case report, chromosome 6p subtelomeric deletions were thought to be a possible underlying cause for periventricular white matter abnormalities [11]. Another study found that the most consistent clinical features in the patients with 6p25 deletion included developmental delay, intellectual disability, language impairment, hearing loss, ophthalmologic, cardiac, and craniofacial abnormalities [12]. In the present case where 6p25 was intact, the patient presented with developmental delay, intellectual disability, and language impairment but had neither hearing loss nor ophthalmologic/cardiac abnormalities.

*C6orf70* is a gene that maps on 6q27 and is one of the deleted genes in this patient (Fig. 3). Haploinsufficiency of *C6orf70* is surmised to play a major role in the pathogenesis of periventricular heterotopia in this patient. Haploinsufficiency or mutation of *C6orf70*, has recently been suggested as the main cause of the brain malformation complex including periventricular heterotopia [13]. *C6orf70* is expressed in developing human brain and is involved in neuronal migration. However, in the previous reports of ring chromosome 6 with 6q27 deletions including *C6orf70*, periventricular heterotopia has not been pointed out [5, 14].

Regarding the white matter abnormality found in this patient, contiguous gene deletions on 6q27 may play a role. Contiguous gene deletion of *PHF10* and *DLL1* could be an underlying mechanism of the white matter abnormality observed in this patient (Fig. 3). Conti *et al.* experimentally demonstrated that down-regulation of *PHF10* and *DLL1* expression in utero delayed neuronal migration in a rodent model [13]. Additionally, *RNASET2*, which is also mapped within the deleted region in the present case (Fig. 3), was reported to have an association with familial cystic leukoencephalopathy [15]. *RNASET2* encodes a putative lysosomal hydrase and the loss of function in zebrafish caused accumulation of amyloid precursor protein in the brain [16].

## Conclusions

This is the first report of periventricular heterotopia and white matter abnormalities manifested in a patient with ring chromosome 6. Further accumulation of cases with similar deletion in this region of chromosome 6 will identify the genes responsible for this neurological phenotype.

## Consent

Written informed consent was obtained from the parents of the patient for publication of this Case report and any accompanying images. A copy of the written consent is available for review by the Editor-in-Chief of this journal.

## Abbreviations

MRI: Magnetic Resonance Image
array-CGH: Microarray-based comparative genomic hybridization
EEG: Electroencephalogram
